# Skin globotriaosylceramide 3 deposits are specific to Fabry disease with classical mutations and associated with small fibre neuropathy

**DOI:** 10.1371/journal.pone.0180581

**Published:** 2017-07-03

**Authors:** Rocco Liguori, Alex Incensi, Silvia de Pasqua, Renzo Mignani, Enrico Fileccia, Marisa Santostefano, Elena Biagini, Claudio Rapezzi, Silvia Palmieri, Ilaria Romani, Walter Borsini, Alessandro Burlina, Roberto Bombardi, Marco Caprini, Patrizia Avoni, Vincenzo Donadio

**Affiliations:** 1 IRCCS Institute of Neurological Sciences, Bologna, Italy; 2 Department of Biomedical and Neuromotor Sciences, University of Bologna, Bologna, Italy; 3 Department of Nephrology, Degli Infermi Hospital, Rimini, Italy; 4 Division of Nephrology, S. Orsola-Malpighi Hospital, Bologna, Italy; 5 Department of Cardiology, S. Orsola-Malpighi Hospital, Bologna, Italy; 6 NEUROFARBA Department, Neuroscience Section, University of Florence, Florence, Italy; 7 Department of Neurology, S. Bassiano Hospital, Bassano del Grappa (VI), Italy; 8 Laboratory of Human and General Physiology, FaBiT, University of Bologna, Bologna, Italy; Weill Cornell Medical College in Qatar, QATAR

## Abstract

**Background:**

Fabry Disease (FD) is characterized by globotriaosylceramide-3 (Gb3) accumulation in several tissues and a small fibre neuropathy (SFN), however the underlying mechanisms are poorly known. This study aimed to: 1) ascertain the presence of Gb3 deposits in skin samples, by an immunofluorescence method collected from FD patients with classical GLA mutations or late-onset FD variants or GLA polymorphisms; 2) correlate skin GB3 deposits with skin innervation.

**Methods:**

we studied 52 genetically-defined FD patients (32 with classical GLA mutations and 20 with late-onset variants or GLA polymorphisms), 15 patients with SFN associated with a specific cause and 22 healthy controls. Subjects underwent skin biopsy to evaluate Gb3 deposits and epi-dermal innervation.

**Results:**

Skin Gb3 deposits were found in all FD patients with classical GLA mutations but never in FD patients with late-onset variants or GLA polymorphisms or in patients with SFN and healthy controls. Abnormal deposits were found inside different skin structures but never inside axons. FD patients with GB3 deposits showed lower skin innervation than FD patients with late-onset variants or polymorphisms.

**Conclusions:**

1) Skin Gb3 deposits are specific to FD patients with classical GLA mutations; 2) Gb3 deposits were associated with lower skin innervation but they were not found inside axons, suggesting an indirect damage on peripheral small fibre innervation.

## Introduction

Fabry Disease (FD) is a rare x-linked disorder[1] characterized by an abnormal function of the lysosomal enzyme alpha-galactosidase A (GLA)[2,3], involved in the cleavage of galactose residuals of globotriaosylceramide-3 (Gb3)[4]. As a consequence, Gb3 starts to accumulate within lysosomes of several tissues involving blood vessels, kidneys, nervous system and heart, as well as dermal fibroblasts[5–8], accounting for FD’s clinical features[9,10] mainly including small fibre neuropathy (SFN)[11], stroke, and renal and cardiac dysfunctions[12]. SFN is mainly responsible for paraesthesias of extremities, hypohydrosis, abdominal pain and diarrhoea[5,11,12] often beginning in childhood with progressive severity throughout life. The available enzyme replacement therapy (ERT) started early before the appearance of organ failure may decrease the pathological impact of this disorder[5].

The possibility to disclose skin Gb3 deposits is interesting because skin is an easily accessible tissue. Skin Gb3 deposits have been demonstrated by using complex techniques such as electron microscopy[13] but they can also be detected by more simple and rapid techniques such as light microscopy[14,15]. However, by using this latter technique skin Gb3 deposits were not found in all analysed patients[15,16], preventing the use of this approach for diagnostic purposes. The reason of the absence of Gb3 deposits in the skin samples of few FD patients is unknown, although a possible correlation with plasma lyso-GL-3 levels was suggested[15]. However, skin Gb3 deposits in classical or late-onset GLA mutations or in healthy controls or patients with SFN of different origin to evaluate their specificity in FD have not been previously studied. In addition, SFN underlying mechanisms in FD are poorly known since contrasting findings were reported in animals[17] and humans[18–20].

Specific aims of this study were: 1) to ascertain the presence of Gb3 deposits in skin samples collected from FD patients with classical GLA mutations or late-onset FD variants or GLA polymorphisms by an immunofluorescence method; and 2) to ascertain the correlation of skin Gb3 deposits with the skin innervation.

## Materials and methods

We studied 52 genetically-defined FD patients including 14 males and 38 females (36±15 vs 45±14 years; p = 0.05) who belong to 26 different families (Tables 1 and 2). Patients were recruited in different FD tertiary centres and evaluated at IRCCS Institute of Neurological Sciences of Bologna. They included 32 patients with classical GLA mutations; 17 patients with late-onset FD variants and 3 patients with GLA polymorphisms, subsequently named as non-classical mutations group (Table 2). To test the specificity of skin Gb3 deposits in FD patients, Gb3 were also searched in 15 patients with SFN associated with specific causes (i.e. 4 patients with diabetes, 3 with Sjogren disease, 2 with hepatitis C, 3 with Parkinson’s disease and 3 with amyotrophic lateral sclerosis) and 22 healthy controls. SFN patients and controls were sex- (5M:10F and 10M:12F respectively) and age-matched (49±8 and 46±18) compared to FD patients (Table 1).

**Table 1:** Clinical and laboratory findings in patients with Fabry disease.

FD pt	Sex	Age	Pain	Skin Gb3	Skin innervation		αGAL activity	ERT
	M:F	years		%	Leg (EFNs/mm)^	Thigh (EFNs/mm)§	%	months
***1***	F	43	4	80	8,8	12	131	6
***2***	M	13	absent	100	7,3	11	100	40
***3***	M	16	absent	100	4	15,5	100	62
***4***	F	21	2	63	16,4	na	134	-
***5***	F	32	absent	75	4,1	4,4	100	-
***6***	M	36	8	100	1,5	3,3	130	3
***7***	F	59	absent	80	4,1	4,7	140	-
***8***	M	17	6	100	7,8	11,6	100	-
***9***	F	19	3	50	8,9	8,3	33,5	-
***10***	F	46	7	50	5,7	12	14	-
***11***	M	37	4	100	7,8	15,4	na	-
***12***	F	46	8	50	7,8	15,4	55	-
***13***	F	27	3	100	9,6	18	84	156
***14***	F	33	absent	100	3,9	19,9	33	-
***15***	F	43	4	50	7,2	9,2	54	-
***16***	F	42	5	50	3	5,5	20,5	-
***17***	F	55	absent	50	5,6	na	na	-
***18***	M	32	8	100	0	na	96,2	96
***19***	M	49	8	100	1	1,75	96	84
***20***	F	62	5	50	6,5	na	59,2	-
***21***	M	32	6	100	2,4	0,6	95,5	39
***22***	F	51	6	75	9,1	10,5	230	52
***23***	F	27	absent	75	6,7	na	49,8	-
***24***	M*	48	absent	100	5	8	82	84
***25***	F	52	absent	75	6	na	200	60
***26***	M	34	4	100	4,2	8	96,8	84
***27***	F	57	absent	75	4,8	9	300	60
***28***	F	51	2	100	2,5	4,4	na	1
***29***	M	25	4	100	5	9,2	96,1	2
***30***	M	49	3	100	0	5	42,3	7
***31***	F	41	absent	25	8	na	180	-
***32***	F	49	2	75	10,6	14,2	na	-
***33***	F	63	absent	0	10,9	14,7	31,3	57
***34***	F	28	7	0	10,9	13,5	62,1	-
***35***	F	31	absent	0	8,75	12,6	47,3	-
***36***	F	36	absent	0	6,9	10,5	67,2	-
***37***	F	28	absent	0	14,6	17,8	108	66
***38***	F	79	6	0	6,1	6,1	na	-
***39***	M*	65	3	0	6,6	6,9	na	7
***40***	M	48	absent	0	5,5	9,8	na	-
***41***	F	46	3	0	6,1	4,8	na	-
***42***	F	41	2	0	8,2	18,4	na	-
***43***	F	30	absent	0	9,9	12,1	na	-
***44***	F	72	10	0	1,5	14,8	na	-
***45***	F	58	3	0	12,6	16	20	-
***46***	F	33	2	0	17,3	21	na	-
***47***	F*	58	absent	0	6	12,7	na	11
***48***	F	42	3	0	11,3	23,7	145	-
***49***	F	60	absent	0	5,9	4,8	126	-
***50***	F	52	6	0	5,1	14,8	na	-
***51***	F	40	absent	0	16,8	25,2	119	-
***52***	F	43	absent	0	8,2	11,3	26	-
mean±SD	**14:38**	**42±15**	**5±2**	**49±43**	**7±4**	**11±6**	**76±71**	**49±41**

Rows 1-32: patients with classical GLA mutations and positive skin Gb3 deposits; Rows 33-52: patients with non-classical GLA mutations and negative skin Gb3 deposits; ERT = enzyme replacement therapy; n.a. = not available; ENFs = number of unmyelinated fibres per linear millimeter of epidermis

* = patients with renal failure

^ = age- and sex-adjusted normative values were taken from Provitera et al., 2016 [32]

§ = laboratory normative values are 24±5 EFNs/mm; cut-off of 12 EFNs/mm for old people (>60 years old) and 27±6 EFNs/mm; cut-off of 14 EFNs/mm for youngest people (<60 years old). We have to use our normative values since no published data are available for the thigh immunofluorescent method.

**Table 2:** Genetic analysis in patients with Fabry disease.

Skin Gb3 deposits					No skin Gb3 deposits				
GLA Mutations	Effect	Type	Pt	Family	GLA Mutations	Effect	Type	Pt	Families
**c.680G>C (R227Q)**	**missense**	**classical**	3	1	**c.644A>G (N215S)**	**missense**	**cardiac variant**[25]	9	4
**c.837G>C (Q279H)**	**missense**	**classical**	3	1	**c.1061T>A (I354K)**	**missense**	**late onset variant**[33]	5	1
**c.861G>A (W287X)**	**deletion**	**classical**	3	1	**c.427G>A (A143T)**	**missense**	**Polymorphism**[34]	2	1
**c.514T>C (C172R)**	**missense**	**classical**	2	1	**c.335G>A (R112H)**	**missense**	**late onset variant**[35]	1	1
**c.1A>G (M1V)**	**missense**	**classical**	2	1	**c.901C>G (R301G)**	**missense**	**cardiac variant**[24]	1	1
**c.2T>C (M1T)**	**missense**	**classical**	2	1	**c.937G>T (D313Y)**	**missense**	**Polymorphism**[36]	1	1
**c.1244T>C (PL415P)**	**missense**	**classical**	1	1	**c.1078G>A (G360S)**	**missense**	**late onset variant**[37]	1	1
**c.611G>T**	**missense**	**classical**	1	1					
**c.835C>A (Q279K)**	**missense**	**classical**	1	1					
**c.1073-1074 insA**	**deletion**	**classical**	4	1					
**c.618_627 delinsA**	**deletion**	**classical**	3	1					
**c.1163-1165 del TCC**	**missense**	**classical**	3	1					
**c.548-3_553del9**	**deletion**	**classical**	1	1					
**c.1006delG**	**deletion**	**classical**	1	1					
**c.1277-1278del AA**	**deletion**	**classical**	1	1					
**c.861-5 C>T (g.9273C>T)**	**deletion**	**classical**	1	1					

GLA = alpha-galactosidase A; Pt = patients

The procedures used were approved by the Human Ethics Committee of Policlinico Sant’Orsola-Malpighi (Bologna) and followed the Helsinki Declaration regarding international clinical research involving human beings. All subjects gave their written informed consent to the study.

### Alpha-GAL activity

Alpha-GAL activity was tested in 39 patients; the remaining 13 patients performed only the genetic analysis because alpha-GAL activity was thought to be a non-diagnostic priority since patients belong to a family with an FD mutation. In addition, this test was performed in several laboratories with different normative values. To obtain a uniform value among different patients, alpha-GAL activity was normalized to the lower cut-off value (i.e., 100% was the lower cut-off value; <100% corresponds to an abnormal value and >100% to a normal value; when alpha-GAL activity value was 0 the normalized value was 0%).

### Clinical examination and laboratory tests

All patients performed a neurological examination. Pain was evaluated by means of a visual analogue scale (VAS), scale marking the level of pain on a 100 mm, non-hatched scale which reported at one end “no pain” and at the other “worst pain imaginable”[20]. A standard motor (tibial nerve bilaterally) and antidromic sensory (sural nerve bilaterally) conduction studies were performed in all patients. Kidney function was evaluated by the glomerular filtration rate (GFR) and considered normal with GFR ≥60 ml/min/1.73 m^2^[21]. Cardiomyopathy was diagnosed in the presence of cardiac fibrosis and left ventricular hypertrophy. Ocular involvement was characterized by corneal changes (”cornea verticillata“) or retinal vessel tortuosity[3].

### Skin biopsy

Three mm punch biopsies were taken from proximal (thigh: 15 cm above the patella) and distal (leg: 10 cm above the lateral malleolus) hairy skin sites. We were unable to take skin samples from the thigh in 6 patients because of patient’s refusal. According to previously published procedures[22] skin samples were immediately fixed in cold Zamboni’s fixative and stored at 4°C overnight.

#### Gb3 deposits

Fifty μm sections were obtained using a freezing sliding microtome (HM550, Thermo Scientific, Walthan, MA, USA) to evaluate Gb3 deposits. Usually 3 free-floating sections were analysed for each skin sample. They were immunostained overnight with a panel of primary antibodies including mouse monoclonal anti-Gb3 (1:1000, TCI chemicals, Portland, Oregon, USA; cat. no. A2506), rabbit pan-neuronal marker protein gene product 9.5 (1:1000; AbD Serotec, Raleigh, NC, USA, cat. no. 7863–0504) and rabbit polyclonal anti-collagen IV (1:500; Novus Biologicals, Littleton, CO, USA; cat. no. NB120-6586). Sections were then washed and secondary antibodies were added for an overnight incubation. As secondary antibodies, an anti-rabbit Alexa Fluor(R) 488 (1:400; Jackson ImmunoResearch, West Grove, PA, USA; cat. no. 711-545-152) or mouse cyanine dye fluorophores 3.18 (1:800; Jackson ImmunoResearch, West Grove, PA, USA; cat. no. 715-165-150) were used. ULEX europaeus (1:100; Vector Laboratories, Burlingame, CA, USA; cat. no. B-1065), a biotinylated endothelium binding lectin, labeled with cyanine dye fluorophore 5.18 coupled with streptavidin (1:400; Jackson ImmunoResearch; cat. no. 016-170-084) was used to show the endothelium. Sections were viewed and analysed under a Nikon confocal microscopy (Eclipse Ti A1). Each image was collected in successive frames of 1–2 μm increments on a Z-stack plan at the appropriate wavelengths for secondary antibodies with a x40 or x60 plan apochromat objective and subsequently projected to obtain a triple-stained 3D digital image by a computerized system. The microscope settings were kept the same for all cases analysed. The analysis was made in a blinded fashion by two authors with expertise in immunoflorescent analysis (DV and IA). Full agreement on the Gb3 staining was reached by the two analysers in all cases without any discordance or uncertain classification (i.e. positive or negative staining in each skin sample analysed). Gb3 staining was rated in each skin site as the percentage of skin structures showing a positive staining at high magnification (x40).

#### Skin innervation

Additional 50 μm sections from the same skin sample were obtained during the freezing sliding microtome session. Three free-floating sections were incubated overnight with a panel of primary antibodies, including the rabbit pan-neuronal marker protein gene product 9.5 (1:1000; AbD Serotec, Raleigh, NC, USA, cat. no. 7863–0504) and mouse collagen IV (ColIV, 1:800, Chemicon, Temecula, CA, USA, cat. no. MAB1910) to define the basal membrane dividing epidermis from dermis. Sections were then washed and secondary antibodies, labeled with mouse Alexa Fluor(R) 488 (1:400; Jackson ImmunoResearch, West Grove, PA, USA, cat. no. 715-545-150) and rabbit cyanine dye fluorophores 3.18 (1:800; Jackson ImmunoResearch, West Grove, PA, USA; cat. no. 711-165-152) were added for an overnight incubation. Epidermal nerve fibre density (ENFs: number of unmyelinated fibres per linear millimetre of epidermis) was calculated by considering a single epidermal nerve fibre marked by PGP 9.5 crossing the dermal–epidermal junction.

### Statistical analysis

We performed statistical analyses using SPSS 15.0 for Windows. Parametric tests were used as Kolmogorov–Smirnov test showed that the variables were normally distributed. Unpaired Student’s t test was used to compare clinical and demographic data between: 1) FD patients vs controls or SFN patients; 2) FD subgroups (classical vs non-classical GLA mutations; males vs females; high vs low skin Gb3 load; ERT vs without ERT). Linear correlation using Pearson test was used to correlate skin Gb3 deposits with clinical parameters or innervation scores. p<0.05 was considered significant.

## Results

### Clinical data

Neurological examination was essentially normal in FD patients except for 2 male patients showing distal tactile sensory loss with a sock distribution. Pain was reported by 21 FD patients, more by males (72%; mean VAS value 50±20) than by females (54%; VAS value 40±20). Pain was more frequent in patients with classical mutations (66% vs 50%). Autonomic symptoms were reported by 22 patients and mainly included palm and sole sweating loss (19 patients) and gastrointestinal dysmotility with nausea, bloating, abdominal cramps or diarrhoea (6) primarily in the group of classical mutations (53% vs 25%). Fifteen patients, mainly those with non-classical mutations (35% vs 20%) had cardiomyopathy. Ten patients (7 with classical and 3 with non-classical mutations) showed groin and umbilicus angiokeratomas. Three patients showed renal failure with decreased GFR (Table 1) and 19 patients, mainly those with classical mutations (50% vs 20%), were on enzyme replacement therapy (ERT). Motor and sensory conduction studies were normal in all patients excluding a peripheral large nerve fibre involvement even in patients with renal failure.

#### Gb3 deposits

Abnormal deposits were found in all patients with classical mutations, whereas all patients with non-classical mutations were negative. FD patients positive for deposits showed a significant young age compared to negative patients (Table 3). No clear difference in alpha-GAL activity was found although this value was not available in 13 patients (3 with classical and 10 with non-classical mutations) making the comparison between these two FD subgroups difficult.

**Table 3:** Comparison between patients with classical (skin Gb3 positive) and non-classical (Gb3 negative) GLA mutations.

	Age	Sex	Skin innervation		αGAL activity
	years	M:F	Leg (EFNs/mm)	Thigh (EFNs/mm)	%
Pt with positive skin Gb3	39±13	12:20	6±3	9±5	73±80
Pt with negative skin Gb3	48±15*	02:18	9±4**	14±5*	84±38

ENFs = number of unmyelinated fibres per linear millimeter of epidermis

positive vs negative p<0.05*; positive vs negative p<0.01**

**Fig 1:**
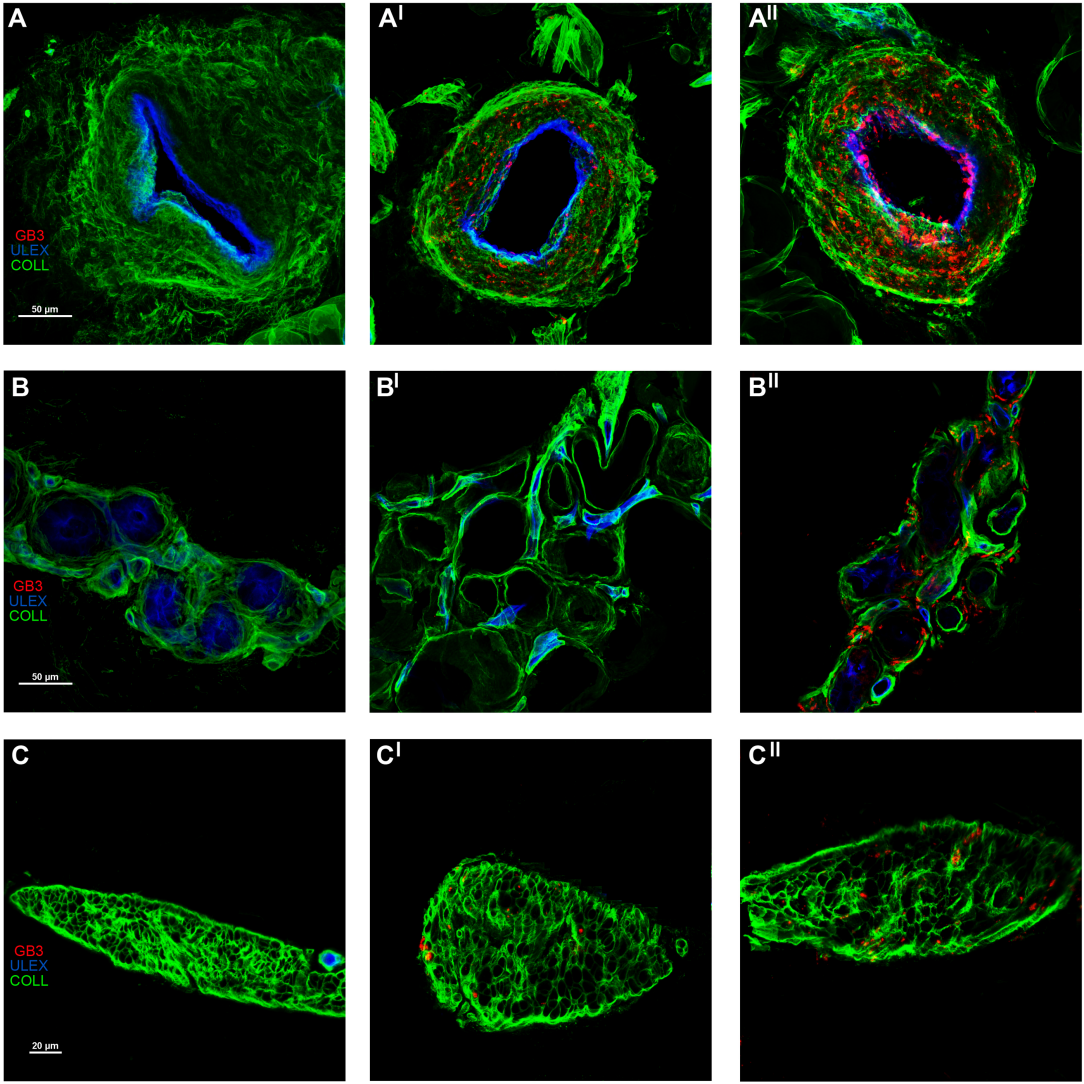
Skin Gb3 deposits in FD patients with classical GLA mutation. Confocal microscope (x40 in A and B; x60 in C) study of Gb3 in skin vessels (A, AI and AII), sweat gland tubules (B, BI and BII) and arrector pilorum muscle (C, CI and CII). A) Skin arterioles showed no Gb3 deposits in the control subject (A). However, these abnormal deposits were abundant in the male FD patient in the wall of the arteriole and its endothelium (AII) and less evident in the female patient (AI); B) Sweat gland tubules of the control showed no Gb3 deposits (B), which were found in the male with FD (BII). The female showed nearly absent Gb3 deposits inside the sweat gland tubules (BI); C) Arrector pilorum muscle cells of the control have no Gb3 deposits (C) but they were found in the male (CII) and less in female FD patient (CI).

Skin Gb3 deposits were found in blood vessel walls and endothelial cells, sweat gland tubules, perineurial, arrector pilorum muscle and dermal cells but never inside the axons (Fig 1). Abnormal deposits were mainly found in males whose skin structures/cells were all affected, whereas females showed abnormal deposits in all the analysed skin vessels but in 95% of muscle arrector pilorum cells, 82% of perineurial cells and 47% of sweat gland tubules. Gb3 were not found in dermal cells of females. No differences were found between thigh and leg regarding the positivity of skin samples and the amount of Gb3 deposits, which were not correlated with age, alpha-GAL activity or skin innervation. However, patients with higher Gb3 deposits (100% of skin annexes affected) showed higher although not significant VAS scale (37±21 vs 28±20; p = 0.3) and autonomic symptoms (67% vs 48%) than patients with lower deposits (62±16% of skin annexes affected). Patients on ERT showed higher Gb3 load compared to patients without ERT (94±10% vs 64±20%, p<0.01) but this finding could be due to a high number of male patients showing diffuse Gb3 deposits (10 on ERT vs 2 without ERT).

#### Epidermal innervation

**Fig 2:**
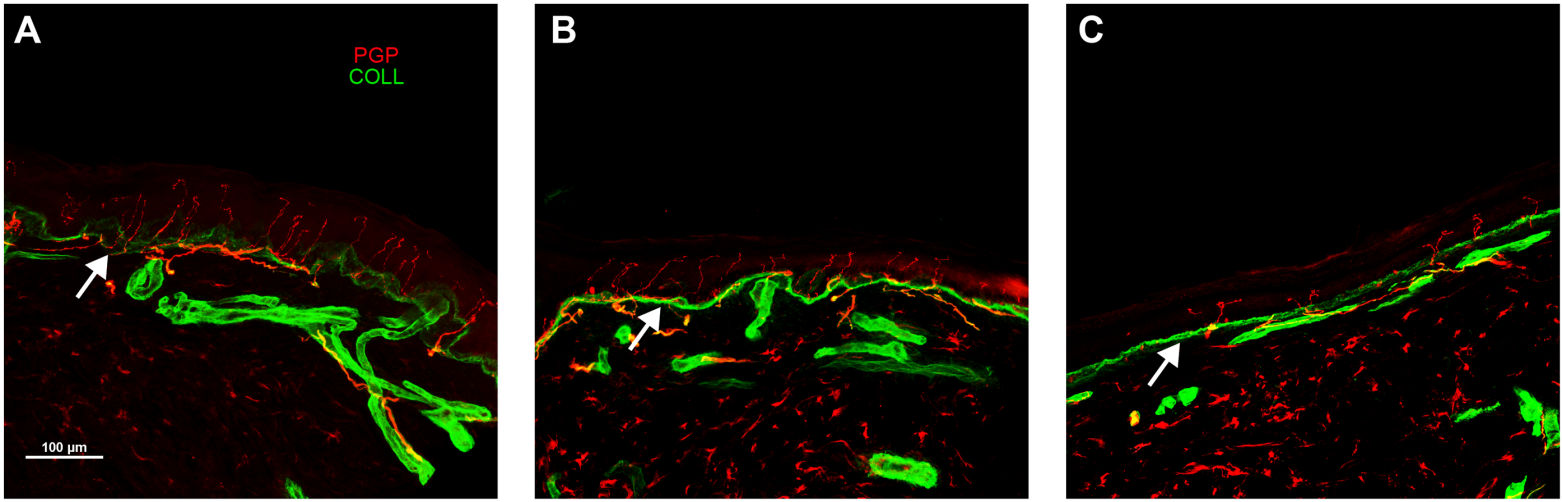
Epidermal nociceptive innervation in FD patients with classical GLA mutation and a control subject. Leg epidermal innervation disclosed by confocal microscope (x40) in an age-matched control subject (A), female (B) and male (C) FD patients. Nerve fibres in red and collagen staining in green. Free-ending PGP immunoreactive nociceptive fibres crossing the dermal–epidermal junction marked by collagen (arrow) are evident in the epidermis of the control (A). FD patients showed a decrease in these fibres, which mainly characterized the male (C) and to a lesser extent the female (B) patient.

ENFs was decreased in FD patients both in the leg (Fig 2) and thigh. The decrease was mainly found in males (4±3 and 8±5; leg and thigh respectively) than in females (8±4 and 13±6), but only the thigh difference was significant (p<0.05). Patients with classical mutations showed lower ENFs than patients with non-classical mutations both in the leg and the thigh (Table 3). Furthermore, SFN was more frequently found in the FD patients with classical mutations (94% vs 70%). Patients on ERT showed lower ENFs both in the leg (p<0.05) and the thigh (p = 0.06) compared to patients without treatment, but the difference was likely due to the prevalence of males in the ERT group.

## Discussion

The main conclusions of our study were: 1) skin Gb3 deposits are specific to FD patients with classical GLA mutations; and 2) Gb3 deposits were associated with lower skin innervation but they were not found inside the axons, suggesting an indirect damage on peripheral small fibre innervation.

FD diagnosis is difficult due to non-specific onset of symptoms. Abnormal skin Gb3 deposits have been studied by complex and expensive techniques involving the use of electron microscopy[13] although a recent study described a simple immunofluorescence technique to disclose Gb3 deposits in FD patients[16]. We demonstrated that this technique showed a promising specificity in identifying FD patients since skin Gb3 deposits were absent in healthy controls and patients with SFN with a specific cause. In addition, our data demonstrated that skin Gb3 staining was associated with classical GLA mutations, whereas FD patients with late-onset FD variants or GLA polymorphisms did not show abnormal Gb3 deposits. These data could be explained by the association between the GLA classical mutations and a lower alpha-GAL activity with a damaging effect on the peripheral nervous system, whereas late-onset FD variants are more often associated with normal alpha-GAL activity, a benign effect on the peripheral nervous system, and a prevalent cardiac involvement[23–25]. As reported earlier, our data did not reliably allow us to evaluate the effect of ERT on skin Gb3 deposits because of a different number of male patients in patients on and without ERT, but also because of a wide variability of this treatment ranging from 1 to 156 months (Table 1). To address this specific aim a focused study before and after ERT treatment in age- and sex-matched patients is required.

SFN is a typical finding in FD[16,20,22] and is responsible for the neuropathic pain complained of by these patients[26]. The mechanism inducing the neuropathic damage is poorly understood. Our data showed that SFN characterized patients displaying a classical mutation but it is more sporadic in patients with non-classical mutations. This finding likely reflects the higher incidence of pain and autonomic symptoms in patients with classical FD mutations and skin Gb3 deposits suggesting the involvement of this abnormal deposits in the pathogenesis of small fibre damage. Indeed, nerve biopsy in Fabry mice showed abnormal accumulation of electrodense vesicles, likely Gb3, within a cluster of unmyelinated axons, which can be responsible of a direct damage to small fibre neurons[17]. By contrast, nerve pathology in FD patients revealed abundant Gb3 deposits in perineurial, endothelial and smooth muscle cells but not within peripheral axons suggesting an indirect effect of Gb3 deposits on peripheral nerve damage by the vasa nervorum obstruction with a subsequent peripheral nerve ischaemia[18–20]. A possible alternative indirect mechanism could be related to abnormalities in spinal ganglia induced by Gb3 deposits in cytoplasmic neurons[27,28].

Our data are in line with these conclusions since we did not disclose Gb3 deposits in skin axons, which were found mainly in the skin vessel walls, sweat gland ducts or arrector pilorum muscle cells. These findings supported an indirect damage of Gb3 on skin’s small nerve fibres. In addition, the ENFs lower value of the leg compared to the thigh regardless of a similar Gb3 deposition could suggest a length-dependent dysfunction as the underlying pathogenetic mechanism affecting small nerve fibres. However, the abnormal expression of ion channels in peripheral nociceptors could be involved in the neuropathic pain in the mouse model of FD also contributing to their structural damage, although the mechanism inducing these abnormalities is not completely understood[29,30].

Finally, the wide range of intrafamilial phenotypic variability regarding the severity of the disease but also the affected target-organs in patients with the same GLA mutation [31] should also be considered analysing the peripheral neuropathy in FD.

Our study has the following limitations: 1) alpha-GAL activity was lacking in several patients preventing us from establishing a reliable correlation with ERT, skin innervation or Gb3 deposits. To this end, an additional study involving a large cohort of patients would be useful; 2) we analysed few male patients with non-classical mutations. An analysis of these patients will be helpful in the future to confirm our current data; 3) furthermore, the quantification of Gb3 deposits by an ultrastructural investigation may increase the accuracy of this analysis.
